# A Novel ShK-Like Toxic Peptide from the Transcriptome of the Cnidarian *Palythoa caribaeorum* Displays Neuroprotection and Cardioprotection in Zebrafish

**DOI:** 10.3390/toxins10060238

**Published:** 2018-06-12

**Authors:** Qiwen Liao, Guiyi Gong, Shirley Weng In Siu, Clarence Tsun Ting Wong, Huidong Yu, Yu Chung Tse, Gandhi Rádis-Baptista, Simon Ming-Yuen Lee

**Affiliations:** 1 State Key Laboratory of Quality Research in Chinese Medicine and Institute of Chinese Medical Sciences, University of Macau, Macau, China; liaoqw2007@126.com (Q.L.); yb67530@connect.umac.mo (G.G.); 2 Department of Computer and Information Science, Faculty of Science and Technology, University of Macau, Macau, China; shirleysiu@umac.mo; 3 Department of Chemistry, The Chinese University of Hong Kong, Shatin, Hong Kong, China; ClarenceTTW@cuhk.edu.hk; 4 Shenzhen Rongxin Biotechnology Co., Ltd., Shenzhen 518054, China; huidong.yu@rongene.com; 5 Department of Biology, South University of Science and Technology of China, Shenzhen 518055, China; tseyc@sustc.edu.cn; 6 Laboratory of Biochemistry and Biotechnology, Institute for Marine Sciences, Federal University of Ceará, Fortaleza 60165-081, Brazil

**Keywords:** zoantharian, ShK-like peptide, dynamics simulation, voltage-gated K^+^ ion channel, calcium-activated K^+^ ion channel, animal toxins

## Abstract

*Palythoa caribaeorum* (class Anthozoa) is a zoantharian which, together with other cnidarians, like jellyfishes, hydra, and sea anemones, possesses specialized structures in its tissues, the cnidocytes, which deliver an array of toxins in order to capture prey and deter predators. The whole transcriptome of *P. caribaeroum* was deep sequenced, and a diversity of toxin-related peptide sequences were identified, and some retrieved for functional analysis. In this work, a peptide precursor containing a ShK domain, named PcShK3, was analyzed by means of computational processing, comprising structural phylogenetic analysis, model prediction, and dynamics simulation of peptide-receptor interaction. The combined data indicated that PcShK3 is a distinct peptide which is homologous to a cluster of peptides belonging to the ShK toxin family. In vivo, PcShK3 distributed across the vitelline membrane and accumulated in the yolk sac stripe of zebrafish larvae. Notably, it displayed a significant cardio-protective effect in zebrafish in concentrations inferior to the IC_50_ (<43.53 ± 6.45 µM), while in high concentrations (>IC_50_), it accumulated in the blood and caused pericardial edema, being cardiotoxic to zebrafish larvae. Remarkably, PcShK3 suppressed the 6-OHDA-induced neurotoxicity on the locomotive behavior of zebrafish. The present results indicated that PcShK3 is a novel member of ShK toxin family, and has the intrinsic ability to induce neuro- and cardio-protective effects or cause cardiac toxicity, according to its effective concentration.

## 1. Introduction

The phylum Cnidaria comprises some of the most venomous known marine animals. Species in this phylum possess unique stinging cells known as cnidocytes, which deliver potent venomous cocktails that immobilize prey for predation or deter them for self-defense [1]. The cnidarian venoms contain disulfide-rich peptides that are cysteine-stabilized toxins which exert their effects by modifying the properties of the ion channels involved in action potential generation in nerve, heart, and skeletal muscle. Collectively, these peptides are typically 10–60 amino acids long, and folded into well-defined secondary structures that are stabilized by multiple, highly-conserved disulfide bonds, which are validated as inhibitors or modulators of different ion channels and neurotransmitter receptors with high potency and selectivity [2]. Based on the number of amino acid residues and disulfide bonds, these toxins can be grouped into four major structural classes: type 1, having 35 to 37 residues with three disulfide bridges; type 2, having 58 to 59 residues and three disulfide bridges; type 3, having 41 to 42 residues and three disulfide bridges; and type 4, having 28 residues and two disulfide bridges. Examples from the first class are BgK from the sea anemone *Bunodosoma granulifera* and ShK from the sea “sun” anemone *Stichodactyla helianthus*.

ShK toxin is a potent blocker of potassium channels. It is a 35-residue peptide originally isolated from sea anemone *Stichodactyla helianthus*. ShK can block not only K_v_1.3, but also other homologous K_v_ subtypes, such as the K_v_1.1, and K_v_3.2 channels [3,4,5]. Owing to the lack of selectivity, ShK is not suitable for use as a therapeutic agent. Instead, by incorporation of non-natural amino acids or organic labels, analogs of ShK have been designed to be selective for K_v_1.3 over K_v_1.1 and other potassium channels. For example, ShK-Dap22 was generated by replacing its Lys-22 into the positively-charged non-natural residue 1,3-diaminopropionic acid (Dap) [4]. ShK-F6CA was a fluorescein-labeled analog of ShK [6]. Both ShK-186 [3] and ShK-192 [7] were created by attaching the phosphor-Tyr and phosphonic-Phe moieties respectively via a hydrophilic linker to Arg-1. Another ShK analog, ShK-K-amide, was generated by adding a Lys residue and an amide at the C-terminus, and was shown to be a potent and selective blocker of K_v_1.3 [8]. A recent study by Peng and colleagues [9] demonstrated that ShK-170, which acted as a K_v_1.3 blocker, could protect mice from radiation-induced brain injury. This result suggested that K_v_ blockers may have neuroprotective effects.

Changes in the intracellular calcium concentration are coupled with changes in membrane potentials. The changes of membrane potentials are integrated by the activation of calcium-activated K^+^ channels (K_Ca_), whose opening probability is increased as a result of membrane hyperpolarization for cytosolic calcium elevation. Many physiological processes, including neurosecretion, smooth muscle tone, action potential shape, and spike frequency adaptation, are regulated by K_Ca_ channels activity. The K_Ca_ channels can be generally categorized into three subfamilies: BK, SK, and IK channels [10]. Indeed, K^+^ channels play an important role in calcium signaling. Wong K. and collaborators found that the opening of K^+^ channels can be considered as one of the mechanisms responsible for the reduction of intracellular calcium concentration in cultured aortic smooth muscle cells [11]. Jow F. and colleagues proposed a coupling between calcium influx and inactivation of voltage-gated A-type K^+^ channels, resulting in membrane depolarization that contributes to after-hyperpolarization [12]. A number of studies have reported that Ca^2+^ influx could be regulated by the voltage-gated K^+^-channel K_v_1.3 and Ca^2+^-activated K^+^ channel K_Ca_3.1, through the calcium-release activated Ca^2+^ channel [13,14,15,16,17,18,19].

Therefore, taking these facts into consideration, sea anemone ShK peptide highlighted the potential of cnidarians to produce valuable drug candidates. Current genomic, transcriptomic, and proteomic studies provided tools to identify novel peptide families [20]. As a zoantharian, a subclass of Hexacorallia, *P. caribaeorum* is a sister species of the sea anemone [21]; herein, we studied a peptide precursor, PcShK3, that contains the canonical ShK domain in its structure. Thus, PcShK3 from *P. caribaeorum* expands the number of ShK analogs, and will hopefully provide a valuable variant to serve as a new template to be developed as a therapeutic candidate. From a functional analysis based on experimental evidence, we found that PcShK3 displayed cardio-protective and neuroprotective activity that, in combination with structurally-guided dissection of peptides which restrained the structure to the central ShK core, confirms our findings on the elucidation of its intrinsic biological and pharmacological activities.

## 2. Results

### 2.1. PcShK3 Is Identified as a Novel ShK-Like Peptide through Molecular Phylogenetic Analysis

**Figure 1:**
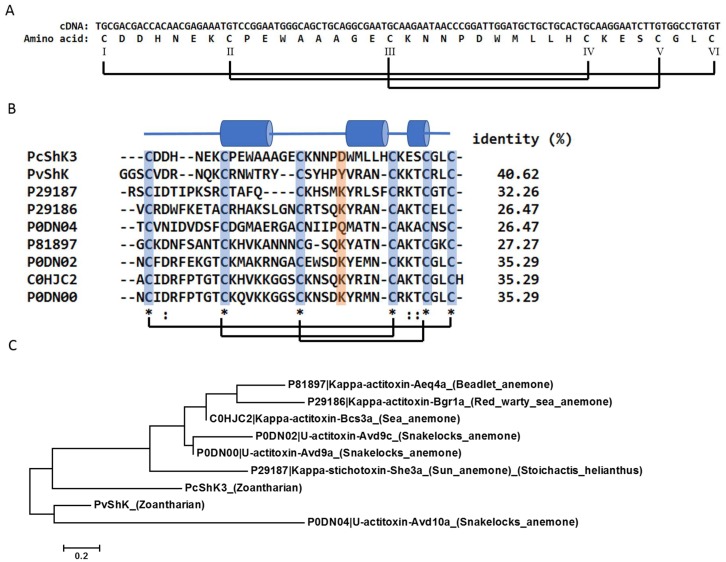
Multiple sequence alignment and phylogenetic analysis of PcShK3 from *P. caribaeorum*. (**A**) cDNA and amino sequence of PcShK3; (**B**) Multiple sequences alignment of *P. caribaeorum* ShK toxin-like peptides and toxins originated from different ShK species of cnidarians (sea anemones). The PcShK3 peptide maintains the cysteine framework, but is distinct in sequence from these sea anemone toxins. Residues highlighted in blue are cysteine. Regions highlighted in orange are residues to block active sites of ion-channels. Cylinders represent α-helices; (**C**) Maximum-likelihood tree from phylogenetic analysis of PcShK3. Notably, except PvShK, the ShK toxin (P29187) originated from *Stichodactyla helianthus* is most similar to PcShK3.

The ShK-like peptide, PcShK3, is structurally similar to other members of the ShK toxin family. It originates from one of our previous studies on the *Palythoa caribaeorum* transcriptome [22], whose sequence was deposited at DDBJ/EMBL/GenBank under the accession number GESO00000000. In this study, the contig was selected for further computational and biological analysis, including primary sequence analysis, structure modeling, and chemical peptide synthesis for in vivo assays and functional studies using zebrafish larvae. Both the cDNA and amino acid sequences of PcShK3 are shown in Figure 1A.

PcShK3 and its homologs from other species of marine organisms were selected for multiple sequence alignment and phylogenetic analysis. From the Maximum-likelihood tree (Figure 1C), it is seen that PcShK3 clusters well with the ShK toxin of *Protopalythoa variabilis*, which was predicted from other zoantharian transcriptomes from our previous study [23]. Also, PcShK3 is phylogenetically related to Kappa-stichotoxin-She3a (UniProt ID: P21987). PcShK3 can be grouped with members of the type 1 sea anemone toxins, each of which contains a cysteine framework similar to that of the ShK toxin from the *Stichodactyla helianthus* sea anemone. They are canonical peptides with 35 to 37 amino acids, containing six highly conserved cysteine residues. Structures of these peptides are thus stabilized by means of three characteristic disulfide-disulfide bonds, namely, C1-C6, C2-C4, C3-C5. The comparison of PcShK3 with other ShK domain-containing toxins in multiple sequence alignment analysis is shown in Figure 1B. As observed, except for the highly conserved cysteine residues and disulfide-disulfide framework, the amino acid sequences are divergent. Close to the *N*-terminal of PcShK3, two amino acids were deleted, as compared to the ShK sequence.

**Figure 2:**
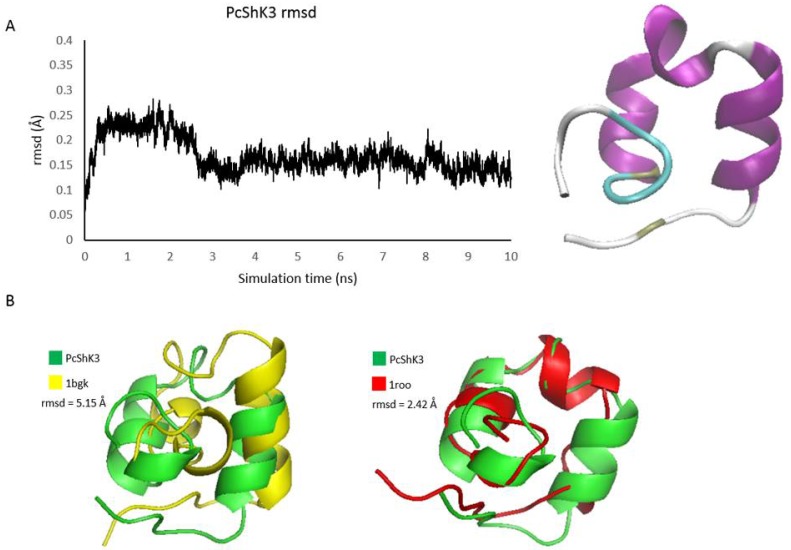
Structure modeling of PcShK3 and structural comparison with ShK and BgK toxins. (**A**) The homology model of PcShK3 was refined by 10 ns simulation after minimization and equilibration steps using GROMACS 5.1; (**B**) Superposition of PcShK3 against crystal structures of ShK toxins, BgK and ShK. The latter was used as template in the homology modeling.

Based on the crystal structure of ShK, the homology model of PcShK3 was obtained. It is a highly stable structure, as shown in the 10-ns molecular dynamics (MD) simulation of the PcShK3 model at solvent. As shown in Figure 2A, the root-mean-squared deviation (rmsd) of the peptide structure reaches a plateau of 0.15 Å after 3 ns, and only light fluctuations were seen in the terminals. Superpositions of the equilibrated PcShK3 structure to BgK (non-template) and ShK (template) exhibit similar characteristics folds, giving rmsd values of 5.15 Å and 2.42 Å, respectively. The first helical segment of PcShK3 highly resembles that of BgK, while the middle, a slightly distorted helix, and the last helical folds, resemble those of ShK (see Figure 2B).

### 2.2. PcShK3 Has the Potential to Block to K_v_1.3 and K_Ca_3.1 through Docking Analysis

**Figure 3:**
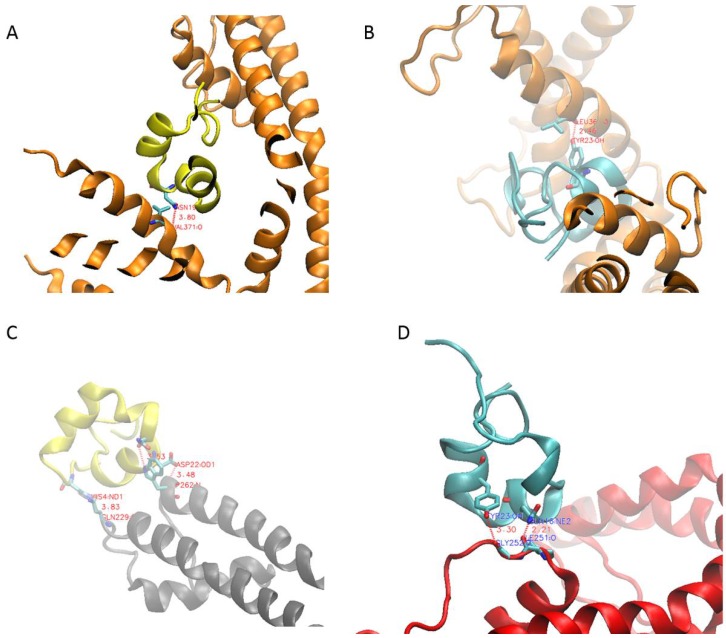
Predicted binding modes of PcShK3 and ShK at the K^+^-channels. (**A**) Interface residues between PcShK3 and K_v_1.3, PcShK3 are shown in yellow; chain D of K_v_1.3 is shown in orange. The binding sites are shown in gray; (**B**) Interface residues between ShK and K_v_1.3, ShK are shown in cyan; chain D of K_v_1.3 is shown in orange; (**C**) Interface residues between PcShK3 and K_Ca_3.1, PcShK3 are shown in yellow; chain C of K_Ca_3.1 is shown in grey; (**D**) Interface residues between ShK and K_Ca_3.1, ShK are shown in cyan, and chain B of K_Ca_3.1 is shown in red.

The well-studied ShK peptide is known to block the activities of both K_v_1.3 and K_Ca_3.1 subtypes of voltage-gated K^+^-ion channels. Electrophysiological studies demonstrated that ShK has a greater affinity for K_v_1.3 (IC_50_ of 133 pM [6,24]) than K_Ca_3.1 (IC50 of 30 nM [5,25]). Since PcShK3 is phylogenetically related to ShK, it is tempting to speculate that PcShK3 may also block these two channels. MD simulation has confirmed the structural stability of the homology model of PcShK3. To gain further insight into potential interactions between this peptide and the channels, protein-protein docking by the ZDOCK server was performed. ZDOCK [26] is an accurate and widely used tool to solve protein-protein docking problems, also for toxin studies [27,28,29]. It has a success rate of 70%, and is ranked among the top 10 methods in the CAPRI benchmark test [30]. After docking was complete, the predicted complexes were then fed into PDBe PISA for interfacial residues analysis. As shown in Figure 3A,B, both PcShK3 and ShK interact with the chain D of K_v_1.3 via one hydrogen bond, namely Asn-19 of PcShK3 to the backbone carbonyl oxygen of the channel residue Val- and Tyr-23 of ShK to the backbone carbonyl oxygen of the channel residue Leu-368. Their computed Gibbs free energy values are also similar. PcShK3 binding yields −30.7 kcal/mol, while ShK binding yields −28.2 kcal/mol. For complexation with K_Ca_3.1, both peptides exploit two residues to interact with the channel, namely Asn-20 and Asp-22, of PcShK3 to the sidechain of the channel residue Trp-262, and Gln-16 and Tyr-23 of ShK to the backbone carbonyl oxygens of the channel residues Ile-251 and Gly-252. The Gibbs free energies of binding in both cases were slightly reduced to −29.7 kcal/mol for PcShK3 and −23.5 kcal/mol for ShK.

### 2.3. PcShK3 Distributed across Vitelline Membrane and Accumulated in the Yolk Sac Stripe of Zebrafish Larvae

To evaluate the biodistribution of the PcShK3, rhodamine B-conjugated PcShK3 was also synthesized to track in vivo how PcShK3 was absorbed and distributed in zebrafish.

**Figure 4:**
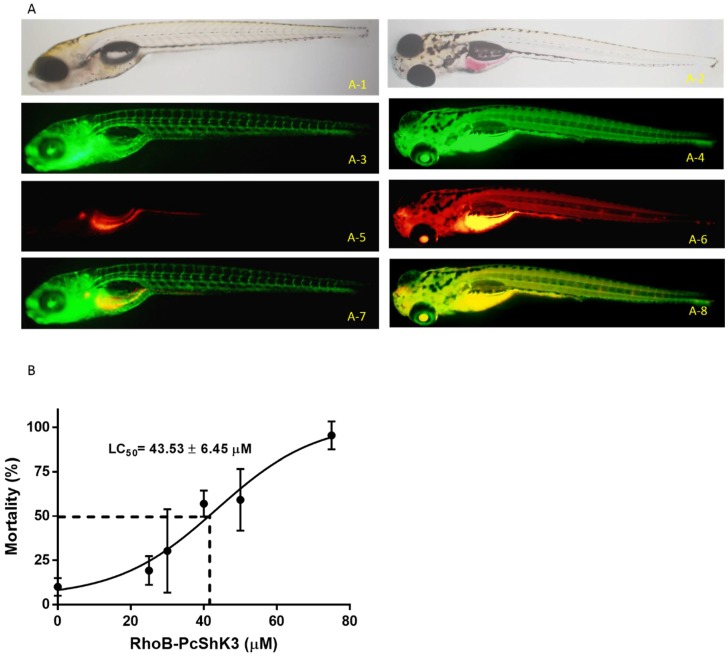
Mortality rate and distribution of *P. caribaeorum* ShK toxin-like peptide. (**A**) Fluorescent images of in vivo distribution of rhodamine B-conjugated peptide in *Tg(fli1:EGFP)y1* zebrafish. Bright field pattern of zebrafish (6 dpf) treated with 20 μM of RhoB-PcShK3 (**A**-**1**) and free rhodamine B as negative control (**A**-**2**) for 24 h. Green fluorescence pattern of EGFP expression in transgenic *Tg(fli1:EGFP)y1* zebrafish (**B**-**3**,**B**-**4**). Fluorescent images (**A**-**5**,**A**-**6**) from zebrafish (6 dpf) after incubation with rhodamine B-conjugated PcShK3 (20 μM) and free rhodamine B (20 μM) for 24 h. Merged images (**A**-**7**) of blood vessels (green) and rhodamine B conjugated PcShK3 (red). Merged fluorescent images (**A**-**8**) of blood vessels (green) and free rhodamine B (red); (**B**) Mortality rate of zebrafish larvae after exposure to peptide. The mortality rate reached 50% when zebrafish larvae was exposed to peptide (40 µM) for 8 h. The mortality rate reached 100% of zebrafish larvae exposed to peptide (75 µM) for 48 h. The LD_50_ is estimated to 43.53 ± 6.45 µM.

In Figure 4A, the biodistribution of PcShK3 is shown, and there is an overlap pattern of peptide distribution and EGFP expression in zebrafish. It showed that the peptide translocated across vitelline membrane and was accumulated in the yolk sac stripe. The assessment of PcShK3′s biological activity (Figure 4B) demonstrated that zebrafish larvae that were exposed to 40 µM of the peptide for 1 h displayed a mortality rate of about 60%. When the peptide concentration reached 75 µM or higher, the lethality was 100% after 48 h of exposure. Therefore, PcShK3, as ShK-like peptide, did not exhibit a high lethal toxicity to zebrafish larvae, with a LD_50_ value fitting in the range of 30 to 40 µM.

### 2.4. PcShK3 Hold the Potential to Improve or Restore the Cardiovascular Function at Lower Concentration

From the docking analysis, we could infer that PcShK3 has the potential to block K_Ca_3.1, a K^+^-dependent calcium ion channel subtype that is widely distributed in cardiovascular system. Then, *Tg(CMLC2:GFP)* zebrafish were utilized to evaluate the pharmacological activity and the protective effect of PcShK3 act on cardiovascular system. The cardiovascular protective effect of the peptide at concentrations lower than 30 µM was evaluated using a set of physiological parameters including heart rate, stroke volume (SV), cardiac output (CO), and fractional shortening (% FS) by means of analyzing the videos and images of the recorded fish hearts. When analyzing the cardiac function parameters of zebrafish larvae exposed to 30 µM, heart rate, SV, % FS, and CO were all decreased. However, these parameters increased overall in a dose-dependent manner at concentrations lower than 20 µM. These findings indicated that our ShK toxin-like peptide derivative, PcShK3, can improve or restore the cardiovascular function at concentrations lower than 20 µM.

**Figure 5:**
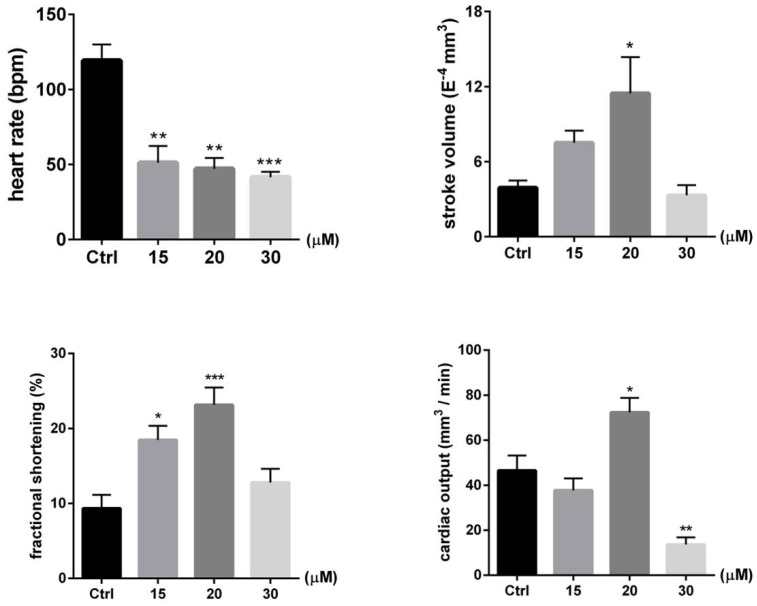
Cardiac functions of zebrafish larvae exposed to various concentrations of PcShK3 peptide for 48 h. Stroke volume (SV), heart rate, cardiac output (CO) and fractional shortening (% FS) of zebrafish were shown. Data are presented as mean ± SEM (n = 10). * *p* < 0.05, ** *p* < 0.01, *** *p* < 0.001 significantly different compared with the control group.

**Figure 6:**
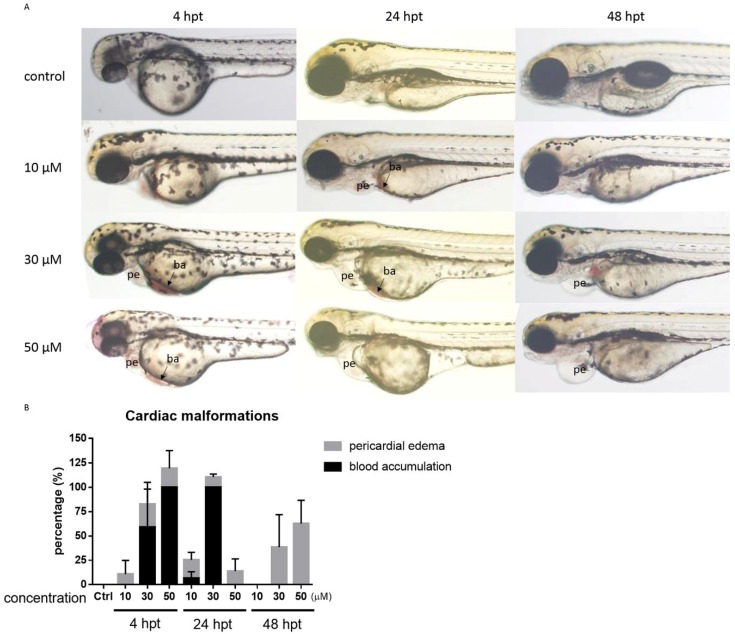
Evaluation of cardiac malformations of zebrafish larvae upon PcShK3 peptide exposure. (**A**) Time and dose-dependent change in developmental cardiac malformations in larvae after exposure to peptide (30 μM) from 4 to 48 hpt. The blood accumulation (ba) appeared after 4 h of drug treatment and decreased after 24 h. The rate of pericardial edema (pe) increased from 24 hpt in a dose-dependent fashion. Values are shown as the mean ± S.D. of three replicates each with 15 larvae; (**B**) Phenotype plot of cardiac abnormalities induced by the peptide.

As seen in Figure 5, the cardiac function parameters were all decreased when zebrafish larvae were exposed to 30 µM of the peptide. Cardiac malformation, which included pericardial edema and blood accumulation, occurred at concentrations higher than 30 µM (Figure 6A). To zebrafish larvae that survived the peptide, i.e., those that were exposed to 30 µM for 4, 24 and 48 h, the phenomena of blood accumulation (ba) and pericardial edema (pe) were observed under fluorescence microscope. The phenotypes of cardiac abnormalities induced by a high dosage of the peptide were evident (Figure 6A). The blood accumulation appeared after 4 h of peptide treatment and decreased after 24 h. The rate of pericardial edema increased from 24 h post-treatment (hpt) in a dose-dependent manner. The cardiac malformation phenotype and graphical plot are shown in Figure 6B.

### 2.5. PcShK3 Could Prevent the In Vivo Dopaminergic (DA) Neuron Loss Induced by 6-Hydroxydopamine (6-OHDA) in Zebrafish

**Figure 7:**
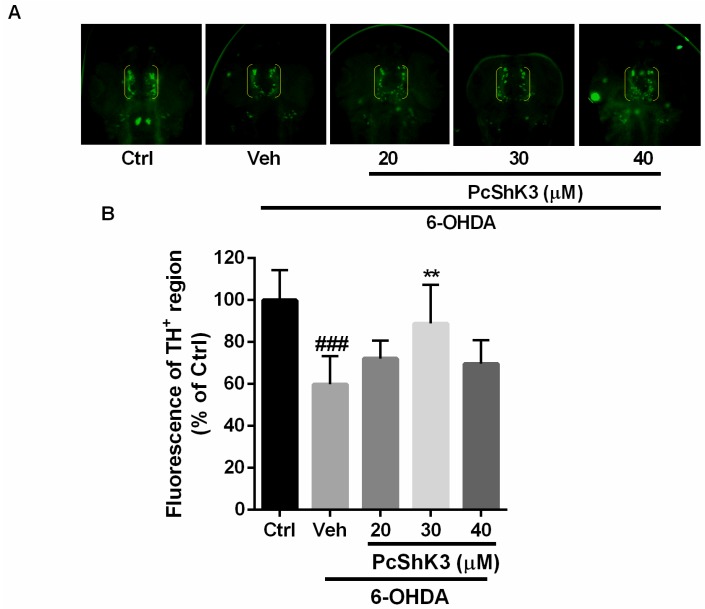
PcShK3 protects against 6-OHDA-induced dopaminergic neuron loss in zebrafish. (**A**) Representative morphology of DA neurons in zebrafish brain indicated by TH staining. TH^+^ neurons in diencephalic region are indicated by yellow brackets; (**B**) quantitative analysis of the area of TH^+^ neurons of each group, ### *p* < 0.001 versus control group, ** *p* < 0.01 versus 6-OHDA group.

To investigate the neuroprotective effect of the peptide, anti-TH whole-mount immunofluorescent staining was used to examine DA neurons in zebrafish larvae. The development of DA neurons in zebrafish is similar to that seen in other vertebrates [31]. As shown in Figure 7, TH-positive neurons in diencephalic area were decreased by about 40% after exposure to 6-OHDA for 48 h (indicated by yellow brackets in Figure 7A and fluorescence of TH^+^ in Figure 7B). Co-treatment with the peptide was able to attenuate DA neuron loss induced by 6-OHDA when the peptide concentration was 30 µM. These results suggested that the peptide provides a protective effect against 6-OHDA-induced dopaminergic neuron death in zebrafish larvae.

**Figure 8:**
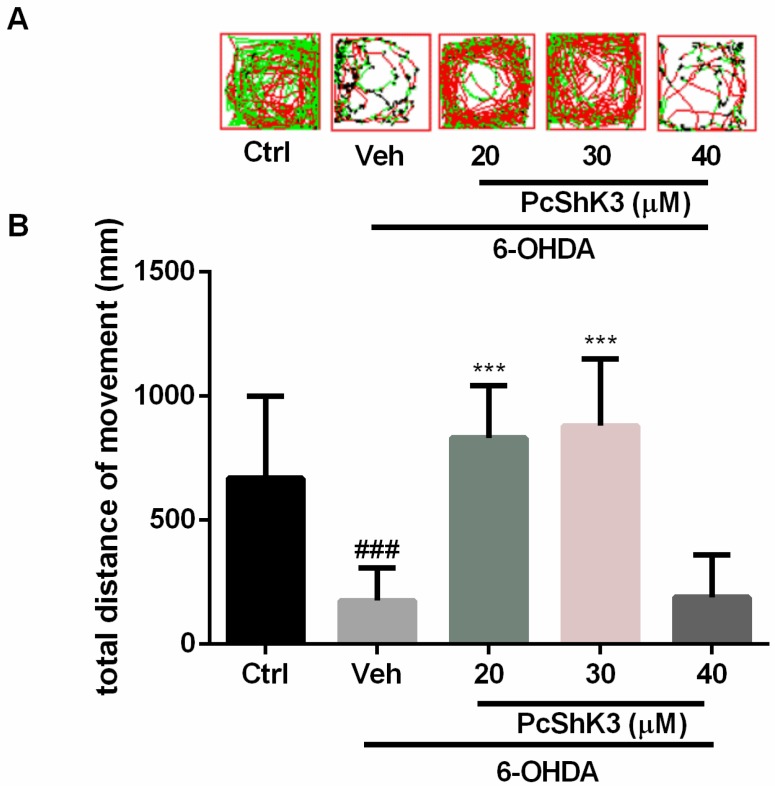
PcShK3 attenuated 6-OHDA-induced lesion in zebrafish. (**A**) Representative patterns of zebrafish swimming track; (**B**) Statistics analysis of total distance moved of different treatment groups, eight fish larvae per group from three independent experiments. ### *p* < 0.005 versus control group, *** *p* < 0.005 versus 6-OHDA group.

In a locomotion test, injury of dopaminergic (DA) neurons by 6-OHDA markedly altered the swimming behavior of zebrafish. As shown in Figure 8, 6-OHDA significantly reduced the swimming distance of the larvae after treatment with 6-OHDA. The 6-OHDA-induced lesion was attenuated in a concentration-dependent manner after co-treatment with PcShK3. These data suggested that PcShK3 can suppress 6-OHDA-induced deficits in the locomotive behavior of zebrafish.

## 3. Discussions

In this study, from the *P. caribaeorum* transcriptome (DDBJ/EMBL/GenBank under the accession GESO00000000), the peptide sequence predicted from the Unigene34015, named PcShK3, was found to have a canonical ShK domain, typical of K_v_1 toxin from sea anemones. From molecular phylogenetic analysis, the homology of PcShK3 was confirmed to share structural similarity with known ShK toxins from many venomous animals, particularly the prototype ShK, and the similar BgK, both originating from distinct species of sea anemone. Moreover, from data of structural modeling and alignments, it was determined that PcShK3 clustered much better with ShK (1roo), rather than with BgK (1 bgk). These sea anemones neurotoxic peptides are known selective inhibitors of K^+^-channel subtypes. In our docking analysis, the Gibbs free energy of complex of ShK and K_v_1.3 is −28.2 kcal/mol. This value is indicative that the peptide interacts with K_v_1.3 through hydrogen bonds between Tyr-23 of ShK and Leu-368 of K_v_1.3, and the distance was calculated to be 2.46 Å. The active site Tyr-23 in this computational output was near Lys-22, like that observed in other experimental studies [32]. Similarly, from the present data, the ShK toxin-like peptide from *P. caribaeorum*, PcShK3, could also bind to K_v_1.3, according to the docking prediction–Asn-19 of PcShK3, part of the binding site, appears to interact to Val-371 of K_v_1.3 through hydrogen bond within 3.80 Å distance, with a Gibbs free energy of −30.7 kcal/mol. The presence of Lys-22 of ShK was believed to protrude into the channel selectivity filter with Asp-499 of K_v_1.3 within 6 Å [29,32,33].

Previous studies showed that ShK toxin displays activity similar to that of cholinesterase inhibitors when injected intraperitoneally in mice [34]. Also, ShK could reduce K^+^ currents in neurons from rat dorsal root ganglia [35]. ShK toxin could induce contractile responses in guinea pig ileum [36]. Interestingly, ShK was able to induce anti-obesity activity and reduction of insulin resistance through the blockade to K_v_1.3 [37,38]. Interestingly, blockers of K_v_1.3 channel in lymphocytes preferentially inhibit the activation of these cells, and therefore show considerable potential as therapeutics for autoimmune diseases such as multiple sclerosis, type 1 diabetes mellitus, and rheumatoid arthritis [39]. It is worthy of note that most studies on the development of ShK toxin analogs as therapeutic agents focus on targeting K_v_1.3 ion channel in autoimmune processes and diseases.

We have tested the *Tg(mpo:GFP)* zebrafish line treated with PcShK3 for immunomodulation response, and found, at a first glance, that PcShK3 was not capable of affecting neutrophil migration (as shown in Figure S1). An important tool for investigating the biodistribution and site of action of a given peptide is the use of fluorescent dyes, which can be covalently linked to the *N*-termini of the peptide sequence. In the survival test, the LD_50_ of the peptide is 43.53 ± 6.45 µM after 48 h of exposure, indicating that the toxicity of PcShK3 derivative in zebrafish is relatively low. However, an evident cardiac malformation was observed in zebrafish larvae upon PcShK3 exposure. In contrast, in 24 h post-treatment (hpt) with the peptide, as seen in Figure 6, blood accumulation and pericardial edema occurred and blood accumulation disappeared at 48 hpt. In other investigative studies with the novel zoanthid ShK-like toxin peptide, identified in the Anthozoa *Protopalyhtoa variabilis*, we have demonstrated a toxic mortality to zebrafish only when peptide concentration was higher than 20 µM [23]. Calcium signaling plays a key role in cardiovascular excitability and function. Excitation-contraction (E-C) coupling in the adult mammalian heart is governed by the Ca^2+^-induced Ca^2+^ release (CICR) mechanism [40]. Our results showed that survival rate of zebrafish larvae is significantly dependent on calcium levels, indicating that the peptide RhoB-PcShK3 probably interacts with the cardiovascular system through K^+^-channel blockade, thus modulating the calcium influx. In fact, the PcShK3 homologous ShK toxin from the *Stichodactyla* sea anemone could also bind to K_Ca_3.1, although the affinity is much less than for other K_v_1 members [3,4,5,25]. The residues Asn-20 and Asp-22 of PcShK3 were docked into Trp-262 of K_Ca_3.1 chain C, respectively. The Gibbs free energy was found to be −29.7 kcal/mol. Therefore, PcShK3 could presumably block K_Ca_3.1 in a similar fashion to ShK.

It is well known that intracellular calcium release from the sarcoplasmic reticulum (SR), through ryanodine receptor, is required for cardiac muscle contraction. The calcium concentration in the cytosol of cardiac myocytes is elevated by approximately 10-fold from a resting level of ~100 nM to ~1 µM with each heart beat [41]. In the cardiomyocyte action potential shape, K^+^-channels open and cause K^+^ ion outflux during phase 3 [42]. Phase 2 is responsible for the large duration of the action potential, and is important in preventing cardiac arrhythmia [42,43]. Therefore, a defect in the removal of calcium from the cytosol during diastole would impair cardiac relaxation.

Some peptide toxins isolated from the animal venom were found to interact with specific targets, and were then have converted into therapeutics. Successful examples of drugs developed from venom peptides include Captopril^®^, a peptidemimetic which was designed based on pentapeptide toxins from the Brazilian viper and which is now used for hypertension treatment [44]; Byetta^®^, an syncretin peptide from the saliva of Gila monster venom, and used as an anti-diabetic agent to treat type 2 diabetes [45]; and Prialt^®^, derived from the MVIIa omega conotoxin specific to block the subtype Ca_v_2.1 of the calcium ion channel, from the predatory cone snail *Conus magus*, and used to treat chronic pain [46,47]. Despite the high potential for drug development, toxicity is a constant concern regarding to the conversion of venom peptides into drug leads and therapeutics.

Interestingly, our findings demonstrate that the novel ShK-like peptide from *P. caribaeorum*, potentially has am intrinsic ability to induce cardiovascular protection and neuroprotection, supposedly through K^+^ channels blockade, since calcium signal modulation was also involved. Evaluation of the cardiac function after exposure of zebrafish to the peptide, at a concentration lower than 20 µM, demonstrated that the heart rate was significantly decreased. Meanwhile, the SV, the % FS, and the CO were increased almost 100% compared to the control group. The hypothesis is that, indeed, PcShK3 targets K_v_ and K_Ca_ channels, and regulates the calcium influx through the membrane of cardiomyocytes, resulting in a specific cardiac protective activity. In addition, some evidence showed that K_v_ blockers can exert a neuroprotective effect. It was found that ShK-170, a K_v_1.3 blocker, could *in vivo* protect against radiation-induced brain injury [9]. Another K^+^-channel blocker, 4-aminopyridine (4AP), could inhibit neuronal cell death through activation of NMDA receptors after blockade to K^+^-channel in the murine hippocampus [48]. Furthermore, it was reported that 4AP could decrease MPTP-induced behavioral lesions. [49] reported that 4-aminopyridine decreases MPTP-induced behavioral lesion. In our study, we found that PcShK3 could suppress 6-OHDA-induced deficits in the locomotive behavior of zebrafish, indicating that PcShK3 has a potential ability to induce neuroprotection, an effect that is useful to ameliorate neurodegenerative disorders. This remarkable finding has provided us with insights to develop novel ShK analogs for prospective application in the research and development of an adjuvant therapy to control cardiovascular dysfunctions and neurodegenerative diseases.

In conclusion, the novel ShK-like peptide PcShK3 from *P. caribaeorum* (a zoantharian species belonging to the subclass Hexacorallia, Cnidaria) has the ability to confer cardiovascular and neurological protective effects in a zebrafish model of drug screening. To further confirm that *P. caribaeorum* ShK-like peptides act as potent ion-channel blockers of the ShK family of toxins, electrophysiological measurements with subtypes of K_v_1 and K_Ca_ channels upon PcShK3 peptide action will be necessary. Moreover, by combining electrophysiology of specific potassium channel subtypes with quantification of intracellular calcium levels, more informative data will give support to hypotheses describing the underlying molecular mechanism of and PcShK3 activity on K_v_1 or K_Ca_. Altogether, the present study reported structural and functional data that provide an insightful perspective to characterize novel ShK-like peptide sequences and their derivatives from zoantharians. Particularly, the peptide displayed an interesting cardio-protective and neuroprotective activity that, in combination with structurally-guided dissection of peptides, can be useful for developing peptide-drug candidates for the investigation and prospective adjuvant treatment of cardiovascular and neurodegenerative diseases.

## 4. Materials and Methods

### 4.1. Primary Sequence Analysis, Structure Modeling and Molecular Dynamics Simulation

The peptide sequences contained ShK domain were downloaded from UniProtKB database. After sequences alignment and editing using the MUSCLE algorithm [50,51], phylogenetic tree was constructed based on the maximum-likelihood method, using the program MEGA version 6 [52]. Reliability of the tree was assessed using 500 bootstrap replicates.

Structures of PcShK3 were modeled using SWISS-MODEL server [53,54], taking the ShK crystal structure as the template [55]. The modeled peptide structure was subjected to energy minimization and molecular dynamics (MD) simulations with CHARMM27 all-atom force field using the GROMACS 5.1 simulation software [56,57]. The equilibrated structure was compared to the known structures of ShK (PDB: 1ROO) and BgK (PDB: 1BGK) toxins. Molecular visualization and structure alignment were achieved using the PyMOL program (version 1.8, Schrödinger, LLC, New York, NY, USA, 2015).

### 4.2. Molecular Docking Analysis

The atomic coordinates of the voltage-gated K^+^-channel subfamily A channels including member 3 (UniProt ID: P22001, K_v_1.3), and intermediate conductance Ca^2+^-activated K^+^-channel protein 4 (UniProt ID: O15554, K_Ca_3.1) were modeled by homology in the SWISS-MODEL server, taking the K_v_1.2 crystal structure (PDB ID: 2R9R) as the template. According to the annotations from UniProt database, ion transport region of K_v_1.3 and K_Ca_3.1 were retained for docking prediction. The Fast Fourier Transform (FFT)-based, initial-stage rigid-body molecular docking algorithm ZDOCK [26] was applied to model the interactions between PcShK3 and the interested channels. PDBe PISA v1.52 [58,59] was utilized for interface residues analysis. The all-structure visualization was achieved using the VMD program v1.9.2 [60].

### 4.3. Peptide Synthesis

Sequences of the PcShK3 peptide were retrieved from the *P. caribaeorum* transcriptome, and synthesized by solid phase chemistry. The presence of a single peak in analytical reverse-phase HPLC (RP-HPLC) and mass spectrometry (MS) analysis was used to confirm a purity grade over 90% (Cellmano Biotech Limited, Hefei, China). Complete deprotection and cleavage were carried out with trifluoroacetic acid in water. The crude peptides were precipitated out by the addition of chilled ether. Finally, the crude peptide was purified by HPLC, freeze-dried for storage, and retested by HPLC to make sure that it qualified (Figure S2A). To track the biodistribution of the peptides, a rhodamine B conjugated PcShK3 was synthesized (Figure S2B). The peptides were solubilized in dimethyl sulfoxide (DMSO) to make a 1 mM stock solution, and diluted in an assay buffer (see composition below) when required. Peptide stock solutions were stored in DMSO at −20 °C.

### 4.4. Zebrafish Maintenance

Transgenic fish lines *Tg(fli1:EGFP)y1* and *Tg(CMLC2:GFP)*, the wild-type AB strain of zebrafish, were manipulated as described in the Zebrafish Handbook [61]. Briefly, the zebrafish embryos were generated by natural pairwise mating (3–12 months old), and were raised at 28.5 °C in embryo medium at 28.5 °C. The ethical approval for the animal experiments was granted by the Animal Research Ethics Committee in University of Macau, China.

### 4.5. Assessment of Survival Rate and Biodistribution of Peptides in Zebrafish Larvae

Zebrafish larvae (*Tg(fli1:EGFP)y1*) at six-day post-fertilization (6-dpf) were exposed to 2-logs (from 5 to 100 µM) of the peptide. The mortality of zebrafish exposed to peptides was determined by observing the presence of heartbeat absence under a light microscope. Zebrafish larvae were separately exposed to a fixed concentration (20 µM) of the peptide for 3 h, then collected and mounted on microscope glass slides. An IX81 motorized inverted fluorescent microscope (Olympus Co., Tokyo, Japan) was used to monitor the biodistribution of the peptide in zebrafish.

### 4.6. Measurement of Morphology and Functions of Zebrafish Heart

The Cell^R imaging system of an IX71 microscope (Olympus) was utilized to evaluate the morphology of the heart and cardiac functions of *Tg(CMLC2:GFP)* zebrafish after exposure to increasing concentration of peptides (10 µM, 30 µM, and 50 µM) for 4 h, 24 h, and 48 h. The zebrafish larvae were placed in 1% agarose to fix in a dorsal orientation. A video segment of each larva was recorded for 10 s (13–15 frames per second) for heart morphology examination. The parameters and morphology of ventricular function of zebrafish were measured, as previously described [62,63]. Briefly, the formula V = 4/3 πab^2^ was used to calculate the volume of ventricles. The longitudinal axis was represented by “a”, while the lateral axis was represented by “b”. Stroke volume (SV) was calculated by end-diastolic volume (EDV) and end-systolic volume (ESV). Cardiac output (CO) was determined by heart rate × stroke volume. Percentage of fractional shortening (% FS) was calculated by the formula FS% = (diastolic diameter − dystolic diameter) / dystolic diameter × 100%.

### 4.7. Anti-Tyrosine Hydroxylase (TH) Whole-Mount Immunostaining

Anti-tyrosine hydroxylase (TH) whole-mount immunostaining of zebrafish was carried out as previously described [64,65]. Briefly, zebrafish embryos at 1 dpf were exposed to 250 µM 6-hydroxydopamine (6-OHDA) with or without the peptides for 2 days. Then the larvae were fixed with 4% paraformaldehyde in PBS for 30 min, rinsed, and stored at −20 °C in absolute methanol. Semi-quantification of TH^+^ cells was assessed by an investigator blinded to the drug treatment history of zebrafish, using ImageJ software [66]. Results were expressed as percentage of area of TH^+^ cells in control group.

### 4.8. Locomotion Behavioral Test

The locomotion test was carried out as described in previous studies [64,65]. Briefly, AB strain zebrafish larvae at 3 dpf were under co-treatment of 250 µM 6-OHDA with various concentrations of the peptides for 4 days; then, zebrafish at 7 dpf were transferred into 96-well plates (1 fish/well). The 96-well plates were put into a Zebrabox and the swimming behavior was monitored by an automated video tracking system (Viewpoint, ZebraLab, LifeSciences, Lyon, France). Before the start of data acquisition, the larvae were settled to allow them to accommodate themselves to the environment in the Zebrabox. The swimming pattern of each fish was recorded in five sessions of 10 min each. The total distance traveled was recorded as the distance that a given zebrafish larva was capable of swimming during the 10 min long session. A statistical analysis of the total distance traveled by each zebrafish larva in the different treatment groups was performed using the ANOVA and Dunnett’s test.

## Supplementary Materials

The following are available online at http://www.mdpi.com/2072-6651/10/6/238/s1. Figure S1: Immunomodulation response assessment of *Tg(mpo:GFP)* zebrafish larvae after PcShK3 treatment, Figure S2: Purification and characterization of the peptides.
