# Supplementary material for: A Novel ShK-Like Toxic Peptide from the Transcriptome of the Cnidarian Palythoa caribaeorum Displays Neuroprotection and Cardioprotection in Zebrafish
Source: Toxins (Basel). 2018 Jun 12;10(6):238. doi: 10.3390/toxins10060238 (PMC6024583; doi:10.3390/toxins10060238)
Supplement: Supplementary file 1 [file toxins-10-00238-s001.pdf]

Supplementary Materials: A Novel ShK-Like Toxic Peptide from the Transcriptome of the Cnidarian *Palythoa caribaeorum* Displays Neuroprotection and Cardioprotection in Zebrafish

Qiwen Liao, Guiyi Gong, Shirley Weng In Siu, Clarence Tsun Ting Wong, Huidong Yu, Yu Chung Tse, Gandhi Rádis-Baptista and Simon Ming-Yuen Lee

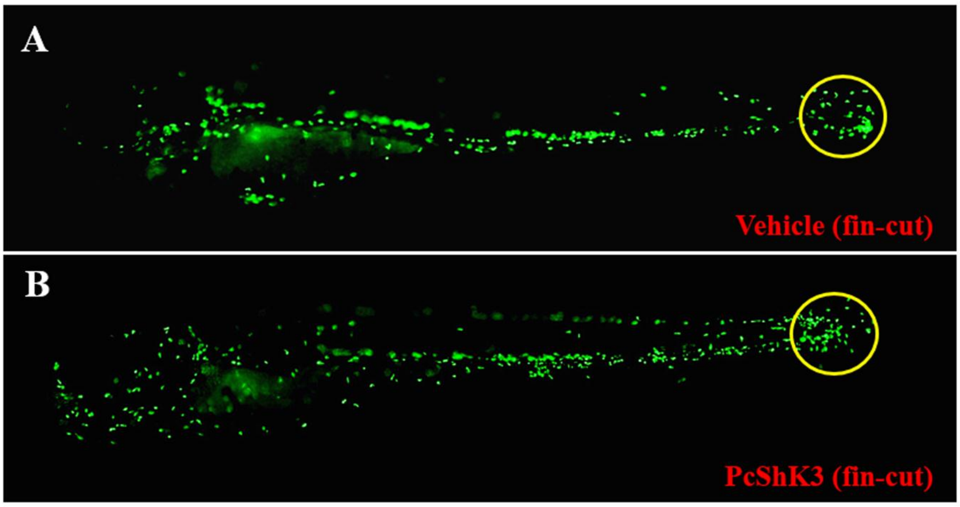

Figure S1. Immunomodulation response assessment of *Tg(mpo:GFP)* zebrafish larvae after PcShK3 treatment. **(A)** Neutrophils (mpo+) accumulated within the fin-cut region; **(B)** No significant changes of neutrophils accumulation could be observed after PcShK3 treatment.

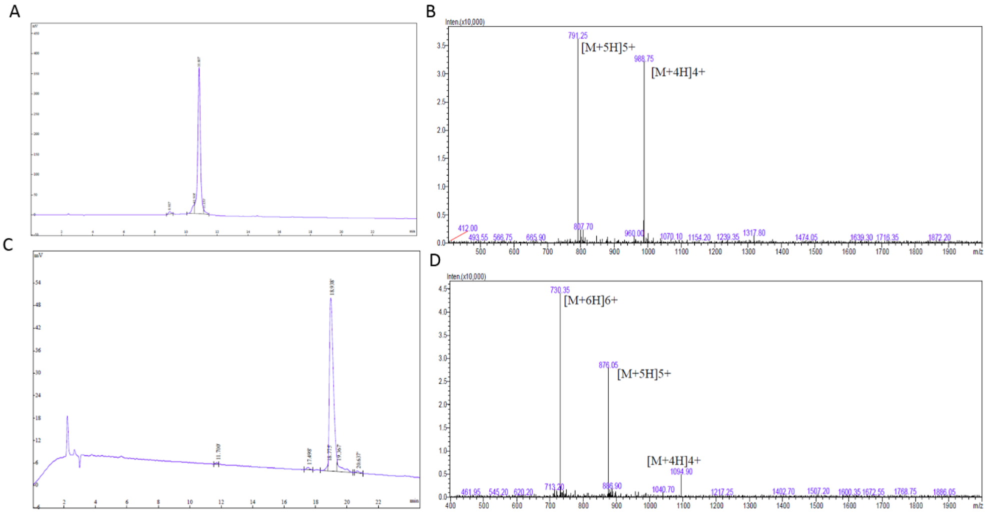

Figure S2. Purification and characterization of the peptides. **(A)** Analytical RP-HPLC chromatograph for the final purified linear PcShK3 peptide with absorbance at 220 nm with purity ≥ 90%. **(B)** ESI-MS analysis of linear PcShK3 peptide. The multicharged ions are deconvoluted to a molecular mass of 3951.45 Da. **(C)** Analytical RP-HPLC chromatograph for the final purified rhodamine B conjugated PcShK3 peptide with absorbance at 220 nm with purity ≥ 90%. **(D)** ESI-MS analysis of rhodamine B conjugated PcShK3 peptide. The multicharged ions are deconvoluted to a molecular mass of 4376.03 Da.
